# Fluorescent carbon-dots enhance light harvesting and photosynthesis by overexpressing *PsbP* and *PsiK* genes

**DOI:** 10.1186/s12951-021-01005-0

**Published:** 2021-08-28

**Authors:** Yuhui Wang, Zhuomi Xie, Xiuhua Wang, Xin Peng, Jianping Zheng

**Affiliations:** 1 Cixi Institute of Biomedical Engineering, Ningbo Institute of Materials Technology and Engineering, Chinese Academy of Sciences, Ningbo, 315300 People’s Republic of China; 2 Ningbo Research Institute of Zhejiang University, Ningbo, 315100 People’s Republic of China; 3 Fujian Agriculture and Forestry University, Fuzhou, 350028 People’s Republic of China

**Keywords:** Fluorescent carbon-dots, Photosynthesis, *N. benthamiana*, *PsbP*, *PsiK*

## Abstract

**Background:**

Fluorescent carbon-dots (CDs) with multifaceted advantages have provided hope for improvement of crop growth. Near infrared (NIR) CDs would be more competitive and promising than short-wavelength emissive CDs, which are not directly utilized by chloroplast. The molecular targets and underlying mechanism of these stimulative effects are rarely mentioned.

**Results:**

NIR-CDs with good mono-dispersity and hydrophily were easily prepared by a one-step microwave-assisted carbonization manner, which showed obvious UV absorptive and far-red emissive properties. The chloroplast-CDs complexes could accelerate the electron transfer from photosystem II (PS II) to photosystem I (PS I). NIR-CDs exhibited a concentration-dependent promotion effect on *N. benthamiana* growth by strengthening photosynthesis. We firstly demonstrated that potential mechanisms behind the photosynthesis-stimulating activity might be related to up-regulated expression of the photosynthesis and chloroplast synthesis related genes, among which *PsbP* and *PsiK* genes are the key regulators.

**Conclusion:**

These results illustrated that NIR-CDs showed great potential in the applications to increase crop yields through ultraviolet light harvesting and elevated photosynthesis efficiency. This work would provide a theoretical basis for the understanding and applications of the luminescent nanomaterials (not limited to CDs) in the sunlight conversion-related sustainable agriculture.

**Graphic abstract:**

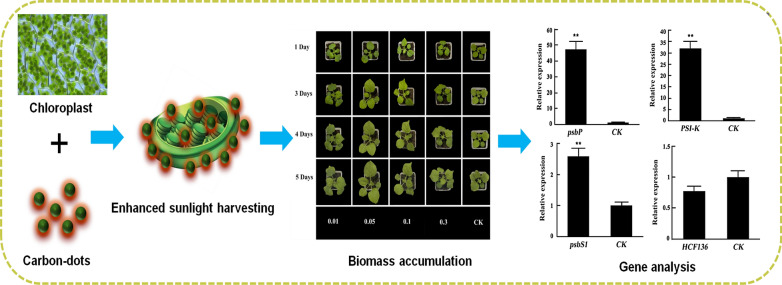

**Supplementary Information:**

The online version contains supplementary material available at 10.1186/s12951-021-01005-0.

## Background

Photosynthesis is one of the most fundamental biochemical processes of organisms, and it is vital to the plant growth on earth. Enhancing photosynthetic efficiency and electron transfer process are thought to be one of the efficient approaches for improving plant growth [1]. Many strategies such as gene regulation [2], genetic improvement [3] and environmental modelling [4] have been applied to improve the photosynthetic efficiency in crops. In the photosynthesis process, sunlight utilization by chloroplasts of plants is limited to visible spectral range [5]. Therefore, it is very promising to build an artificial hybrid photosynthesis system to improve the ability of plants to capture and convert solar energy efficiently [6].

Luminescent nanomaterials (LNMs) have been paid more attention in many fields like chemo/biosensing, bioimaging, catalysis and nanomedicine due to their unique photophysical properties [7]. As light conversion agents, LNMs would have the potential to enhance sunlight harvesting and promote photosynthesis efficiency [8]. In previous reports, semiconductor quantum dots (SQDs) [9], silicon quantum dots [10], lanthanide-doped up-conversion phosphors [11] and noble metal nanoclusters [12] were exploited on the plant growth and development. However, toxicity of the heavy metal ions, high cost and low fluorescence quantum yield greatly limit their further applications [13, 14]. Therefore, it is meaningful and challenging to seek new LNMs with the features of low cost, good biocompatibility and outstanding optical properties [15].

Recently, fluorescent carbon-dots (CDs) emerging as a new class of inorganic phosphors have attracted increasing attention [16–19]. Due to their obvious merits of facile and cheap preparation, excellent fluorescence characters, CDs have demonstrated many promising applications such as chemo/biosensing, bioimaging, catalysis, and optoelectronic devices [20–24]. Specially, most of the reported CDs are no/low toxic and biocompatible, and have been demonstrated great potential in biological-relevant fields [25]. However, some CDs with positive charge reveal cyto and geno-toxicity in some degree [26]. The biosafety of the longtime exposure to CDs remains to be a challenging issue, and should be taken into consideration [27]. The development of CDs in photosynthesis has become a hot research topic [28–33]. For instance, Chandra et al. reported a blue-emissive CDs-chloroplasts hybrid nanosystem to accelerate electron transfer from CDs to chloroplast [34]. Li et al. reported a strategy to enhance photosynthetic efficiency via light-harvesting with dual-emissive CDs [35]. Nonetheless, most of the CDs show short-wavelength blue/green emission, which is not directly utilized by chloroplast. To overcome the above issue, far-red emissive CDs with high quantum yield and good water-solubility were synthesized, and have demonstrated to be efficient in the enhancement of plant growth and photosynthesis [36]. Meanwhile, former researches have presented that CDs were no phytotoxicity on the growth of plants, and could be transferred from the roots to the stems and leaves through the vascular system [37]. The above reports confirmed that CDs would be competitive and promising agents to enhance photosynthesis or improve growth of crops. Nonetheless, the underlying mechanisms of these stimulative effects were hardly mentioned. There are few reports about the influence of CDs on the expression level of genes and the plant quality. [8]

Herein, in order to understand the mechanism of CDs-caused enhancive photosynthesis efficiency, representative near-infrared (NIR) emissive CDs (a model light-conversion phosphors) and *Nicotiana* benthamiana (*N.* *benthamiana*, taken as a plant model) were preferentially selected, respectively. The NIR-CDs were verified to be effective in the enhancement of ultraviolet light absorption and the electron transfer from CDs to the chloroplasts *N.* *benthamiana*. So, improved photosynthetic efficiency was observed both in vivo and in vitro. Furthermore, some photosynthetic and chloroplast synthesis-relevant genes were inspected. The results demonstrated that *PsbP* and *PsiK* genes were the key regulators of the photosynthesis-stimulating effect that activated by the NIR-CDs. To the best of our knowledge, this is the first mechanism research on the genes level, which provides a manner to study the mechanism of LNMs-induced enhanced sunlight absorption and photosynthesis efficiency. In addition, this work would provide a theoretical basis for the understanding and applications of other luminescent nanomaterials (not limited to CDs) in the sunlight conversion-related sustainable agriculture.

## Results

### Characterizations of the CDs

**Fig. 1:**
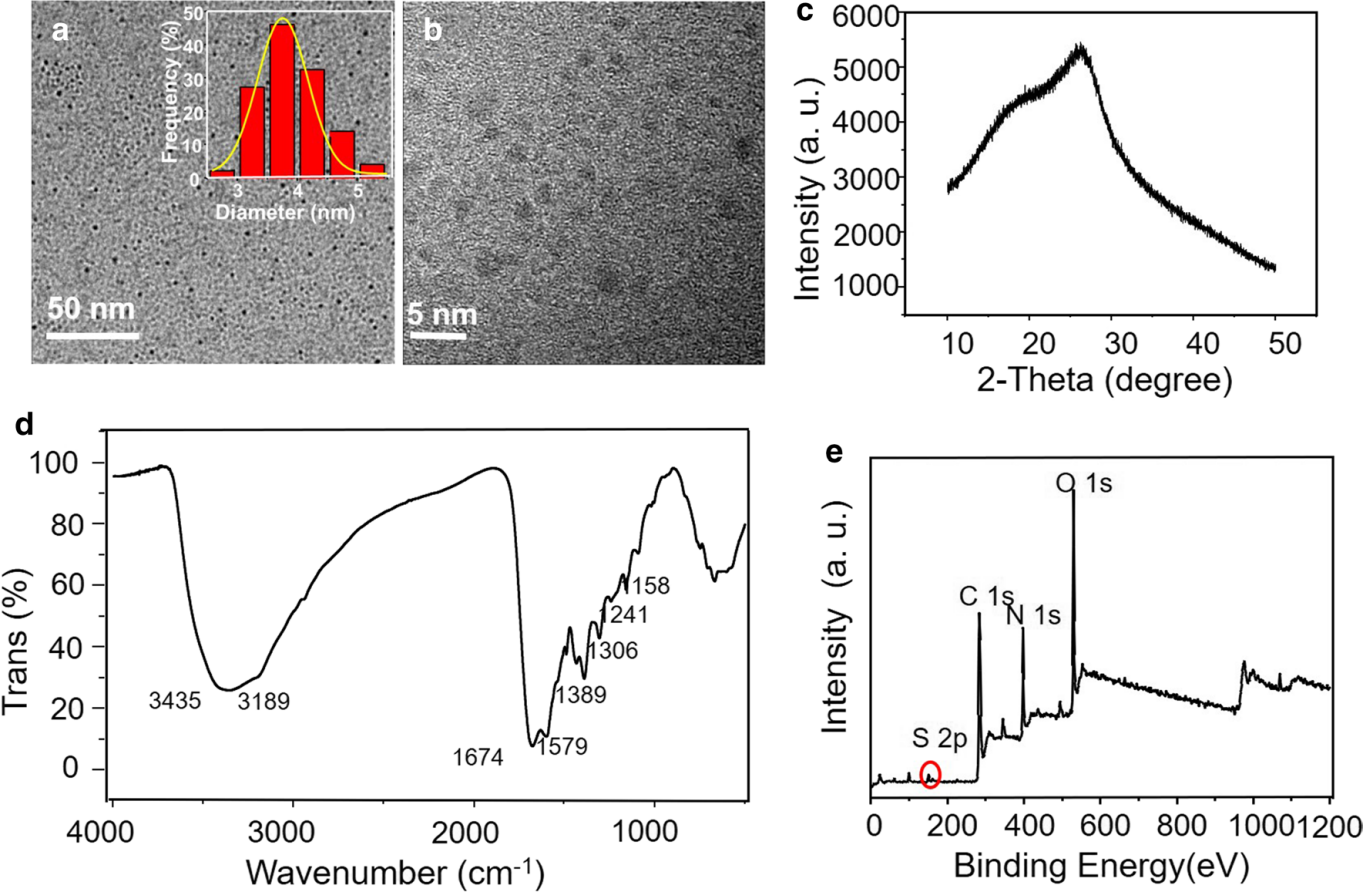
**a** TEM image of the NIR-CDs (inset: the corresponding TEM histogram and Gauss fitting of particle size distribution). HR-TEM (**b**), XRD pattern (**c**), FT-IR spectrum (**d**) and XPS (**e**) measurements of the NIR-CDs

The NIR-CDs were facilely synthesized by a microwave-assisted carbonization method using glutathione and formamide as the raw materials. To characterize the morphology, structure and surface state of the prepared CDs, TEM, XRD, FT-IR, XPS, Raman and Zeta potential measurements were performed. As shown in Fig. 1a, the harvested NIR-CDs exhibit uniform and spherical morphologies with narrow size distribution and an average diameter of 3.8 nm. However, no obvious lattice fringes are observed in the HR-TEM image (Fig. 1b), implying that they are mostly noncrystalline. A typical peak at 26° [(002) plane] in the XRD pattern (Fig. 1c) further verifies the noncrystalline graphite structure of the CDs [38]. Typical Raman spectrum (Additional file 1: Figure S1) also confirms the graphite nature of the NIR-CDs. The distinct peaks at 1557 and 1321 cm^−1^ represent the typical G-band and D-band, respectively. Meanwhile, a low ratio of D to G strongly attests the existence of pristine carbon in the NIR-CDs [39]. In Fig. 1d, a broad and strong absorption band from 3000 to 3700 cm^−1^ with two peaks centered at 3435 and 3189 cm^−1^ is clearly observed, which is attributed to the stretching vibrations of O–H and N–H, demonstrating the existence and abundance of hydrophilic hydroxyl and amino groups. The peaks at 1674, 1579, 1389 cm^−1^ belong to the stretching vibrations of the C=O, C=C/C=N, and C–N bonds, respectively. The peaks at 1158 and 1241 cm^−1^ are attributed to C–O and C–N stretching vibrations. The absorption band at 1000–1100 cm^−1^ is attributed to C=S and oxidized S bonds [40, 41]. These FT-IR assignments are clearly verified by XPS analysis (Fig. 1e). Representative peaks of C 1 s, N 1 s, O 1 s, and S 2p are observed at 283, 397, 529, 161 eV, respectively, which indicates that the CDs mainly contain C, N, O and S elements (atom ration, C:O:N:S = 63.61:16.77:19.28:0.34). High resolution C 1 s spectrum (Additional file 1: Figure S2a) shows five peaks at 284.8, 286.3, 288, 289.4 and 291.2 eV, corresponding to C=C/C−C, C−N, C−O, C=N/C=O, and N−C=O, respectively. Three unique peaks of pyridine-like N, amino N, and pyrrolelike N at 398.6, 400.1, and 402.5 eV are observed in high resolution N 1 s spectrum (Additional file 1: Figure S2b). O 1 s XPS spectrum exhibit the typical peaks of C−OH and C=O at 531.5 and 533.7 eV, respectively (Additional file 1: Figure S2c). In addition, the higher solution S 2p spectrum (Additional file 1: Figure S2d) can be fitted with four binding energies of 162.2, 163.6, 164.7 and 168.7, which are assigned to thiolate, 2p_3/2_ and 2p_1/2_ of thiophene S, and oxidized S, respectively [42, 43]. Zeta potential measurement reveals that the NIR-CDs are negatively charged (ζ = − 15.8 mV, Additional file 1: Figure S3), which would enable strong electrostatic exclusion and colloid stability.

**Fig. 2:**
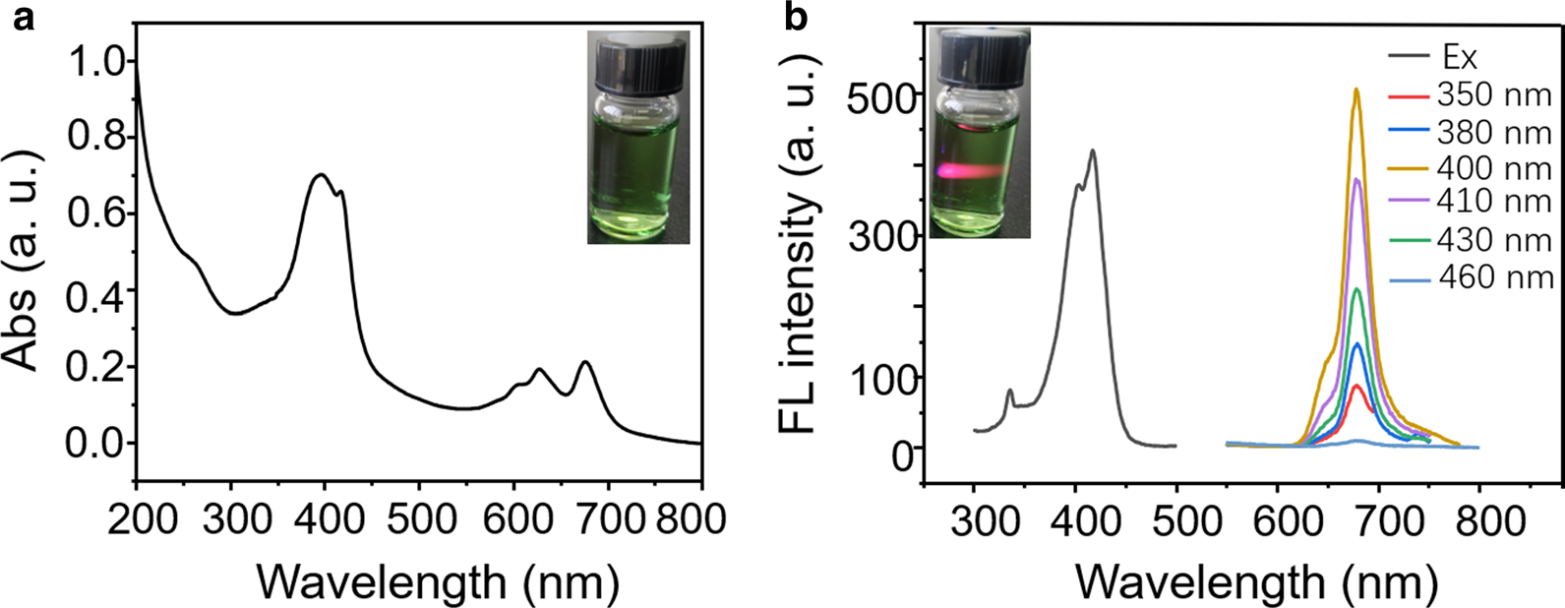
**a** UV–vis absorption spectrum of the NIR-CDs dissolved in water (100 µg/mL), inset: digital photograph of the NIR-CDs solution under daylight. **b** Fluorescence excitation spectrum (black line) and emission spectra under varying excitation wavelengths of the NIR-CDs solution (10 µg/mL), inset: digital photograph of the NIR-CDs under the excitation of laser pointer (405 nm)

Next, the photophysical properties of the NIR-CDs were investigated in detail. Figure 2a represents the UV–Vis absorption spectrum. The NIR-CDs present three main absorption bands i.e., 240–300 nm, 350–450 nm, and 550–750 nm, which are generally assigned to the typical π → π* transition of the aromatic C=C bond, π → π* and n → π* transitions of the aromatic π system containing the C=O, C=N, and C=S bonds, respectively [44]. As shown in Fig. 2b, the CDs display brightly deep-red emission from 625 to 710 nm with a sharp peak centered at 680 nm. And an excitation-independent fluorescence emission property of the NIR-CDs is observed distinctly. The fluorescence excitation spectrum indicates that the excitation focuses on blue spectrum range with the optimal excitation wavelength of 420 nm. Moreover, the averaged lifetime is measured and calculated to be 2.8 ns with bi-exponential decays (Additional file 1: Figure S4), and the absolute fluorescence quantum yield of the NIR-CDs is measured to be 17.8% under the optimal excitation i.e., 420 nm. In addition, photostability of the NIR-CDs was estimated. In Additional file 1: Figure S5, the emission intensities slightly decrease upon the irradiation of ultraviolet lamp, implying good tolerance to photobleaching of the NIR-CDs. The CDs solution keeps very stable fluorescence emission even after one month storage at ambient environment (Additional file 1: Figure S6). So, the above unique optical properties e.g., strong absorption in the ultraviolet-blue region, efficient and stable emission in far-red region make this NIR-CDs promising agent in light-harvesting and electron transfer from photosystem II (PS II) to photosystem I (PS I) in chloroplasts.

### NIR-CDs treatment significantly promoted the growth and development of *N. benthamiana*

**Fig. 3:**
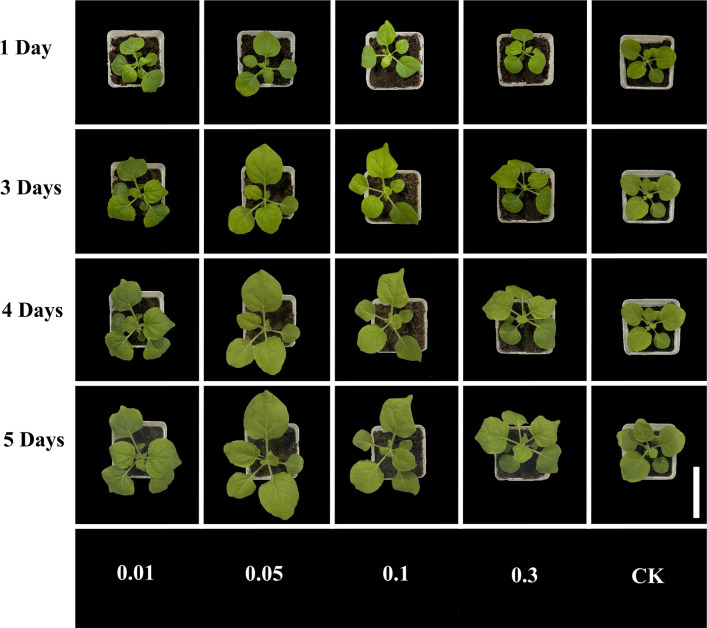
Effect of different concentrations of NIR-CDs (0–0.3 mg/mL) on the growth of *N.* *benthamiana* during 5 days (scale bar: 10 cm)

*N.benthamiana* were cultured with different concentrations of NIR-CDs solution (experiment group) and pure water (control group), respectively. Figure 3 is the photograph of *N.* *benthamiana* exposed to different concentrations of NIR-CDs during 5 days. As shown in it, there is an obvious difference of growth vigour of *N.* *benthamiana* between the control group and CDs-treated groups. A concentration-dependent promotion effect on *N.* *benthamiana* growth was found in the concentration range of NIR-CDs from 0 to 0.1 mg/mL. It turned out that a concentration threshold (0.3 mg/mL) was presented, above which the differences were not significant. 0.05 mg/mL of the CDs was the optimum concentration.

**Fig. 4:**
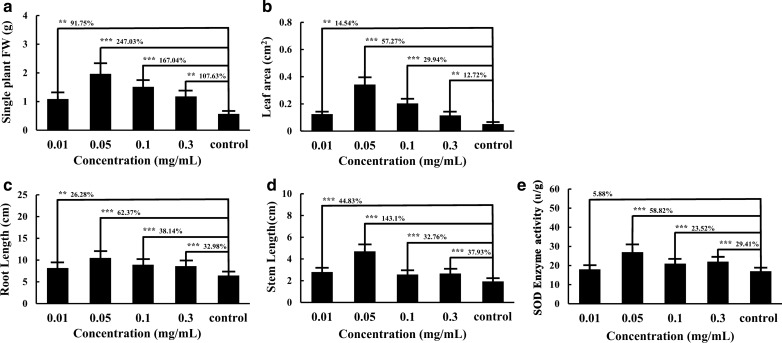
Growth potential indexes of different concentrations of NIR-CDs (0–0.3 mg/mL) treatment after 5 days, compared to the control. **a** Single plant fresh weight (FW) of *N. benthamiana*. **b** Leaf area. **c** Root length. **d** Stem length. **e** SOD activity. All the experiments were repeated three times at least. Marked with *P < 0.05, **P < 0.01 and ***P < 0.001 exhibit significant differences from control, respectively

In order to obtain accurate results, root length, stem length, leaf area and biomass were measured at least three times, respectively. As shown in Fig. 4, it could be found that both the stem and root elongation depended on the concentration of NIR-CDs. The stem length of *N.* *benthamiana* exposed to the CDs (0.01 mg/mL, 0.05 mg/mL, 0.1 mg/mL and 0.3 mg/mL) was longer 44.83 ± 3.9%, 143.1 ± 9.8%, 32.76 ± 4.1% and 37.93 ± 3.7% than the control, respectively. As for the elongation of root length, they were 26.28 ± 3.4%, 62.37 ± 6.8%, 38.14 ± 4.5% and 32.98 ± 4.3%, respectively. The leaf area exposed to CDs (0.01 mg/mL, 0.05 mg/mL, 0.1 mg/mL and 0.3 mg/mL) was increased by 14.54 ± 2.2%, 57.27 ± 4.6%, 29.94 ± 2.5%, and 12.72 ± 0.9% than the control, respectively. When the concentration was 0.3 mg/mL, NIR-CDs didn't stimulate the growth of *N. benthamiana* anymore. 0.05 mg/mL of CDs improved root length, stem length and biomass prominently, which was the optimal concentration for the growth of *N. benthamiana*, with a growth rate of single plant fresh weight of 247.03 ± 26%. To further evaluate the metabolic activity of *N. benthamiana* under the treatment of different concentrations of NIR-CDs, the SOD activity was also investigated with the results shown in Fig. 4e. CDs at 0.01 mg/mL, 0.05 mg/mL, 0.1 mg/mL, and 0.3 mg/mL significantly increased SOD activity by 5.88 ± 0.6%, 58.82 ± 5.9%, 23.52 ± 3.2% and 29.41 ± 3.2% respectively, as compared to the control group.

### Process of uptake and transmission of NIR-CDs in *N. benthamiana*

**Fig. 5:**
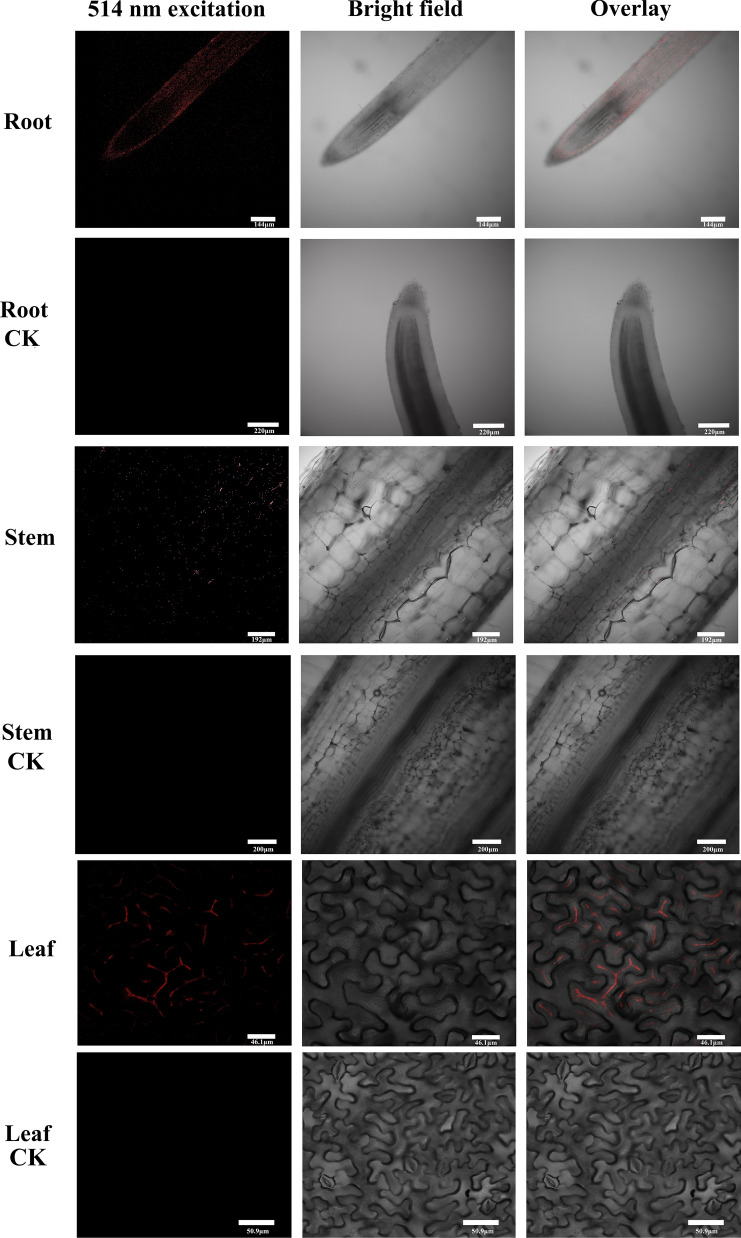
Laser scanning microscopy (LSM) images of root, stem, and leave cultured with NIR-CDs (0.05 mg/mL) for 5 days. All images were collected under the same exposure conditions

In order to study the uptake and translocation conditions of NIR-CDs in *N. benthamiana* during the growth stage, confocal images of root, stem, and leaf were displayed using Laser-scanning confocal fluorescence microscope after 5 days incubation with 0.05 mg/mL CDs. As shown in Fig. 5, the uptake of NIR-CDs by *N. benthamiana* could be identified in vivo due to the red luminescent emissions from CDs observed under 514 nm excitation. The bright field images were also investigated and overlain with luminescence images. In contrast, no red luminescence and any autofluorescence background of tissue were detected in the control group. The distribution of luminescence signals of CDs predominantly existed in the roots, and less in stems and leaves, revealing that CDs were absorbed by root and transported to the stems and leaves.

The confocal images also revealed that the CDs could penetrate cell wall into vascular bundle system. Thus, the growth-promoting effect of CDs might be due to the entry of CDs into plant cells to regulate plant physiological activities.

### NIR-CDs treatment significantly improved chlorophyll content and photosynthesis

**Fig. 6:**
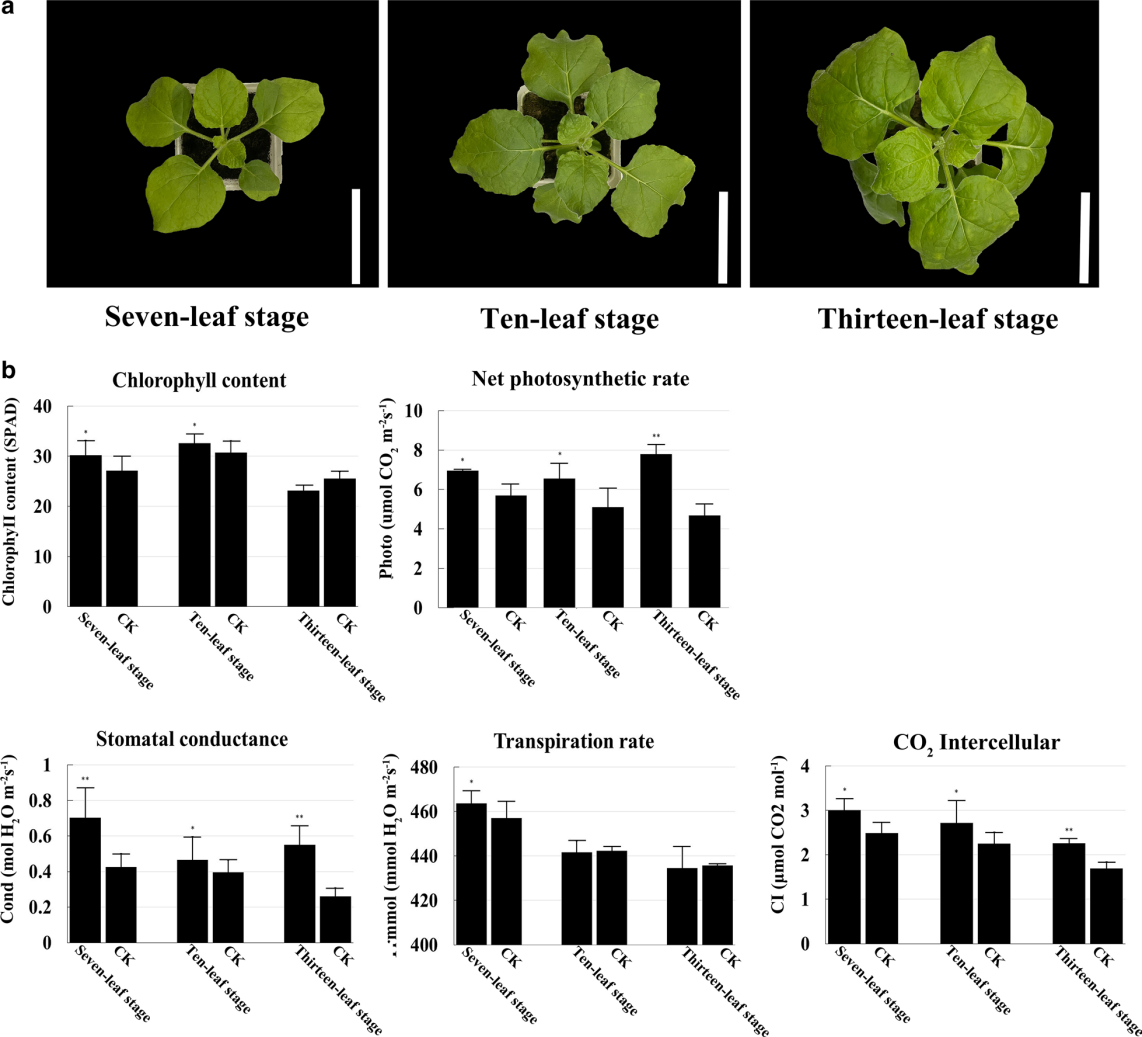
The phenotype and photosynthetic parameters of *N. benthamiana* seedlings exposed to 0.05 mg/mL of NIR-CDs in seven-leaf, ten-leaf, and thirteen-leaf stage respectively. **a** The phenotype of *N. benthamiana* seedlings growth (scale bar: 10 cm). **b** The chlorophyll content, net photosynthetic rate, intercellular CO_2_ concentration, transpiration rate and stomatal conductance of *N. benthamiana* seedlings. Marked with *P < 0.05, **P < 0.01 exhibit significant differences from control, respectively

The effect of NIR-CDs treatment on photosynthesis was determined. 0.05 mg/mL of NIR-CDs enhanced chlorophyll content by 11.4%, 6.08%, and 10.21% in seven-leaf, ten-leaf, and thirteen-leaf stage, respectively (*P* < 0.05). CDs treatment induced significant increase in net photosynthetic rate at all the three stages, and the maximum increase (66.68%) was achieved by 0.05 mg/mL of CDs in thirteen-leaf stage. The rate of photosynthesis, which is evaluated by CO_2_ entry through the stomata and fixation within the chloroplast, can change when plants are subjected to various treatments. Exposure to CDs at 0.05 mg/mL significantly increased the transpiration rate by 0.15–1.47%, stomatal conductance by 17.5–110.8% (*P* < 0.01) and intercellular CO_2_ by 20.38–33.82% (*P* < 0.01) as compared to the control group (Fig. 6).

### Growth-promoting effect of NIR-CDs were achieved by upregulating expression level of the genes involved in photosynthesis

**Fig. 7:**
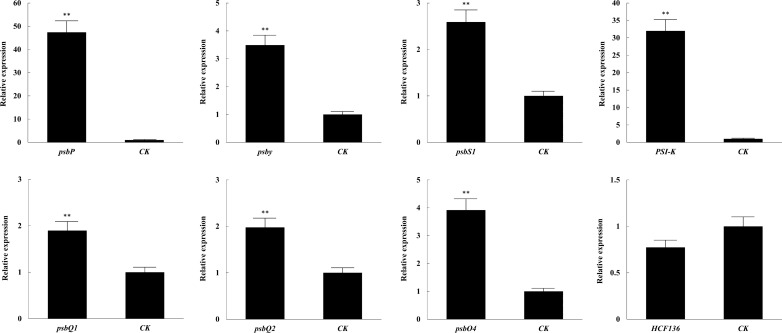
The relative transcript level of eight major photosynthetic genes exposure to 0.05 mg/mL NIR-CDs after 5 days duration of incubation. Marked with *P < 0.05 **P < 0.01 exhibit significant differences from the control, respectively

The increased growth rate might be due to NIR-CDs stimulating the photosynthesis of *N. benthamiana* seedlings. To prove the conjecture, we investigated the effects of NIR-CDs on the gene expression level, which were involved in photosynthesis. In this work, eight major photosynthetic genes (*Psi-K*, *PsbP*, *PsbS1*, *PsbY*, *HCF136*, *PsbQ1*, *PsbQ2*, and *PsbO4*) of *N. benthamiana* were examined after 5 days incubation with 0.05 mg/mL NIR-CDs. The primers of these candidate genes and reference gene are displayed in Table 1. The results shown in Fig. 7 indicated that the CDs could activate significantly the overexpression of seven *N. benthamiana* photosynthetic genes compared with control group. Furthermore, the expression levels of *PsbP* and *Psi-K* genes were increased the most by 48-fold and 32-fold as compared to the control, respectively. It revealed that *PsbP* and *Psi-K* genes were most sensitive to the stimulation effect of NIR-CDs. At the concentration of 0.05 mg/mL NIR-CDs, the expression levels of the five genes (*PsbS1*, *PsbY*, *PsbQ1*, *PsbQ2*, and *PsbO4*) were significantly increased by 148.3%, 246.7%, 90.3%, 98.4%, and 294.4%, respectively after incubation of 5 days. In addition, there were no significant differences in the expression level of *HCF136*, which suggested NIR-CDs had no significant effects on *HCF* family genes. These results coincided with the response of the chlorophyll content and photosynthetic rate.

**Table 1:** Primer sequences of the genes involved in photosynthesis used for qRT-PCR analysis

Gene ID	Length (bp)	Primer-F (5′–3′)	Primer-R (5′–3′)
*PsbP*	138	GCTCTCACTGTCCTCATT	GAATCCATCTCCGTTGTATG
*PsbS1*	124	CTATGAAGCAGAGCCACTA	AGCCTTATCAAGACCAGTAG
*Psi-K*	121	CCATCAGCAAACAGGAAG	GACCAACAACACCACAAG
*PsbQ1*	129	TCGTCTCAGAGCAGAATAC	GCATGGTCCAGATCACTA
*PsbQ2*	112	ACCGTCATCTCTGCTAAG	GGCTGTTCTTGGTCTTTG
*PsbO4*	174	GTTCCTTGTGCCATCATAC	CTCAGCGTGATCTTACCT
*PsbY*	153	GACATAGCAGAAGGAGACA	ACCAGTAAGACCAAGACC
*HCF136*	103	GCATTCTTATGTCGGCTAC	GTCACGAATCCATGTCTTG
*GAPDH*	125	AGCTCAAGGGAATTCTCGATG	AACCTTAACCATGTCATCTCCC

## Discussion

### Enhanced sunlight harvesting and photosynthesis efficiency of the CDs- chloroplast hybrids in vitro

**Fig. 8:**
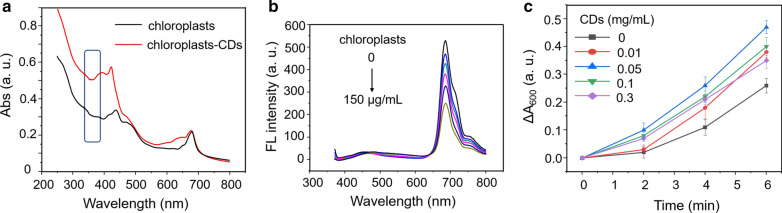
**a** Absorption spectra of chloroplast suspension (100 µg/mL) and the chloroplast-CDs complex (NIR-CDs: 50 µg/mL). **b** Fluorescence emission spectra (excitation wavelength: 380 nm) of the CDs (5 µg/mL) in the presence of different amounts of chloroplast (0, 5, 10, 15, 20 and 30 µg/mL). **c** DCPIP reductions upon the addition of varying concentrations of NIR-CDs (0, 0.01, 0.05, 0.1 and 0.3 mg/mL) in the CDs-chloroplast complexes under the light intensity of 4 mW/cm^2^

Generally, far-red radiation can promote the growth of plant [45]. Herein, in order to verify the NIR-CDs-induced enhanced photosynthesis, interaction between the CDs and chloroplast, and the classical Hill reaction were severally studied. In Fig. 8a, the isolated chloroplast suspension shows two obvious absorption bands i.e., 400–500 and 650–700 nm. But, after being covered with the CDs, the absorption of chloroplast-CDs complexes is broadened and enhanced remarkably, especially in UV region (350–400 nm), which is mainly attributed to the typical UV absorption property of NIR-CDs (Fig. 2a). So, the hybrid chloroplast-CDs photosystem would be much more efficient in the harvesting of solar light. As shown in Fig. 8b, fluorescence emissions of the CDs gradually decrease along with the constant addition of chloroplast, implying relatively strong adsorption between each other. Considering good spectrum overlap (650–700 nm) between fluorescence emission of the NIR-CDs and absorption of chloroplast, we speculate that the reason for the above fluorescence quenching is the occurrence of energy transfer from the CDs to chloroplast [36, 46].

As mentioned above, the NIR-CDs possess an obvious UV absorption feature, and their fluorescence emissions overlap well with the absorption of chloroplasts in the far-red region. So, it is possible and desirable that the NIR-CDs can absorb and transform rarely-used UV radiation to highly utilized far-red light to enhance photosynthesis efficiency. To authenticate this hypothesis, a typical Hill reaction was performed. Hill reaction provide a facile method to research the light-dependent transfer of electrons by chloroplasts in photosynthesis that results in the cleavage of water molecules and liberation of oxygen [47, 48]. Owing to the strong capacity of electron capture, 2,6-dichlorophenolindophenol (DCPIP) is routinely used as an indicator to monitor the electrons transfer from PS II to PS I during the photoreaction process. The absorption alterations at 600 nm indicate the reduction of DCPIP. By this way, the photosynthesis rate was estimated through measuring the absorption change of DCPIP. In this study, a xenon lamp was adopted as the light source. As shown in Fig. 8c, upon the durative exposure, the absorption of DCPIP decreases obviously, suggesting the emergence of photosynthesis of the subjected chloroplasts. Compared with the isolated chloroplasts, all the complexes of NIR-CDs-chloroplast can accelerate the reduction of DCPIP, and absorb and transform more UV light to far-red light that can be directly utilized by chloroplast. Thus, in the hybrid photosystem, the adoption of NIR-CDs is favorable for the light harvesting and utilization of chloroplasts in photosynthesis. Moreover, it is clearly to observe that the optimum concentration of the NIR-CDs is 50 μg/mL for the achievement of the highest photosynthesis rate. The above results obviously demonstrate that the proposed NIR-CDs show enhanced light harvesting in UV region, and their near infrared emissions would further promote the electrons transfer process from PS II to PS I, thereby improving the photosynthesis efficiency in vitro.

### NIR-CDs strengthen photosynthesis by overexpressing PsbP and PsiK genes, promoting photosynthetic electron transfer and activating PSII and PSI in *N. benthamiana*

Photosynthesis is the key reaction to sustain life. In higher plants, oxygenic photosynthesis takes place in chloroplasts, in which protein complexes involved in the light-harvesting and photosynthetic electron transport are located in thylakoid membranes. Two light-energy driven photosystems (PS), PSI and PSII, are key components of photosynthetic electron transport machinery [49].

Photosystem I (PSI) is a supramolecular complex consisting of 17 different polypeptides located in the higher plant membranes. PSI-K is a subunit of photosystem I, and is significant for organizing the peripheral light-harvesting complexes on the core antenna of PSI [50]. In the present study, the expression levels of *PsiK* gene were dramatically increased by 32-fold as compared to the control*.* The results suggested that *PsiK* also played a key role in response to the growth-promoting effect of NIR-CDs.

Molecular oxygen metabolism is vital for photosynthesis, which is generated mainly by photosystem II (PS II). Photosystem II (PSII) is a light-driven water–plastoquinone oxidoreductase, in which the oxygen-evolving complex (OEC) catalyzes the water-splitting reaction [51].

It is known that some membrane-extrinsic subunits associated to the lumenal side of PSII in higher plants, including *PsbO*, *PsbP*, *PsbQ*, and *PsbR*, play crucial roles in optimizing water-oxidizing activity [52]. Some studies about *PsbP*-lacking transgenic plants suggested that *PsbP* was essential for full PSII function and chloroplast development [53]. In *PsbP*-deficient *N. tabacum*, PSII was hypersensitive to light and rapidly inactivated when the repair process of damaged PSII was inhibited. Moreover, the manganese cluster of *PsbP*-deficient leaves was markedly unstable. Another study on *A. thaliana* where both *PsbQ* genes (psbQ-1 and psbQ-2) had been suppressed, demonstrated that *PsbQ* was indispensable for photoautotrophy under low-light stress [54]. Kakiuchi et al. confirmed that *PsbQ* could significantly compensate for functional defects of mutated *PsbPs*, suggesting that *PsbQ* had a role in stabilizing the functional binding of *PsbP* in higher plant PSII [51].

*PsbP* and *PsbQ* were shown to play different roles in PSII of plants, including the likelihood that *PsbP* was in closer association to the oxygen-evolving catalytic center, *PsbQ* might not be required for PSII assembly, but should be involved in stabilizing the binding of *PsbP* [49]. Suppression of *PsbP* might lead to a reduction in growth rate and a wide range of defects in PSII function, such as lower quantum yield, lower oxygen-evolving activity, and a slower electron transfer rate at the donor side of PSII [55]. However, the suppression of *PsbQ* did not result in the severe malfunction of PSII in *Arabidopsis* [54]. These results suggested that *PsbQ* might play a less important role in PSII of higher plant than *PsbP*.

Similar to these previous studies, in the present study, the expression levels of *PsbP* gene were increased the most by 48-fold as compared to the control, and it was far higher than the expression levels of *PsbQ1* and *PsbQ2.* The results also indicated that *PsbP*, *PsbQ1,* and *PsbQ2* were the regulatory genes involved in PSII in response to the stimulation effect of NIR-CDs, especially *PsbP* was the dominant regulator for the growth-promoting effect of NIR-CDs.

A study on high chlorophyll fluorescence (*HCF*) photosynthetic mutants of Arabidopsis led to the identification of HCF136. It was a nucleus-encoded assembly factor, which was essential for assembly or stability of the PSII reaction center complex, and might function as a chaperone-like assembly factor. *HCF136* was also reported to be required for efficient repair of PSII in Synechocystis [56]. On the contrary, another *HCF136* homologue was reported to be needless for assembly of the PSII, and hence was not required for photoautotrophic growth of *Synechococcus* PCC 7002 [57]. In the present study, no significant changes in the expression level of *HCF136* gene were observed in response to the stimulating photosynthesis effect of NIR-CDs, which also indicated that *HCF136* was not closely associated with photoautotrophy of *N. benthamiana.*

Previously, far-red emissive CDs (FR-CDs) have been synthesized and applied for enhanced sunlight absorption and photosynthesis efficiency [36]. The as-prepared FR-CDs was an efficient converter transferring ultraviolet A (UV-A) light to 625−800 nm far-red emission, which could be directly absorbed and utilized by chloroplasts. The in vivo experiment demonstrated 51.14% enhancement of fresh weights compared with that of the control group. Compared with the FR-CDs, the proposed NIR-CDs showed similar absorption range, but relatively narrow emission band (625–720 nm). Due to the better spectrum overlap between the NIR-CDs emission and the absorption of chloroplast (640–710 nm), much more photons were absorbed and transferred to chloroplast, and thereby improving the photosynthesis [58]. Aa a result, the enhancement of fresh weight reached to 247.03%, nearly five times than that of FR-CDs-caused weight enhancement. Moreover, in this work, it is the first report on the mechanism research in the molecular level of genes. It would provide a manner to study the interaction mechanism between luminescent nanomaterials and photosynthesis-related genes. A detailed comparison on optical properties, fresh weight enhancement, toxicity and mechanism between this work and previous reports is summarized in Additional file 1: Table S1. As shown therein, the proposed NIR-CDs show overall advantages compared to the previous luminescent nanomaterials.

## Conclusion

In summary, NIR emissive CDs were successfully adopted for enhanced photosynthesis both in vitro and in vivo, and the activation mechanism was further illustrated by gene analysis. The CDs with good mono-dispersity and hydrophily were easily prepared by a one-step microwave-assisted carbonization manner. The obtained NIR-CDs showed obvious absorption in the UV region, strong and stable far-red fluorescence emission. The hybridized chloroplast-CDs complexes revealed effective absorption in UV region and accelerated the electron transfer from photosystem II (PS II) to photosystem I (PS I). NIR-CDs exhibited a concentration-dependent promotion effect on *N. benthamiana* growth, and it was achieved by strengthening photosynthesis. Seven photosynthetic genes and chloroplast synthesis related genes were demonstrated to be closely related to the photosynthesis-stimulating effect, among which *PsbP* and *PsiK* genes were the key regulators. The results in this work revealed the underlying molecular responders in response to NIR-CDs treatment. This result could provide a theoretical basis for expanding the applications of nanomaterial in sustainable agriculture practices.

## Materials and methods

### Materials and apparatus

Reduced glutathione, Na_2_HPO_4_, KH_2_PO_4_ and KCl were obtained from Aladdin Chemistry Co., Ltd (Shanghai, China). Sucrose, formamide and 2,6-dichlorophenolindophenol (DCPIP) were purchased from Sinopharm Chemical Reagent Co., Ltd. (Shanghai, China). All reagents were used as received without further purification. All aqueous solutions were prepared using deionized (DI) water.

A microwave oven [Galanz, P70F20CL-DG(B0)] was employed for the synthesis of CDs. The sizes and morphologies of the CDs were characterized by high resolution transmission electron microscopy (HR-TEM) (Tecnai F20) with an acceleration voltage of 200 kV. Fourier transform infrared (FT-IR) spectrum was performed on a Nicolet 6700 FT-IR spectrometer via the KBr pellet method. X-ray photoelectron spectroscopy (XPS) measurements were performed on a ESCALAB 250Xi (Thermo Scientific). The crystal phase of NIR-CDs was identified by a Bruker D8 Discover X-ray diffractometer (XRD) with 2θ range from 10° to 50° at a scanning rate of 4°/min, with Cu Ka irradiation (k = 1.5406 Å). Raman spectrum was recorded on a Renishaw inVia Raman spectrophotometer using 532 nm laser as the excitation resource. Fluorescence excitation and emission spectra were recorded on a Perkin Elmer spectrophotometer (LS-55). UV–Vis absorption were obtained from a Agilent Cary 300 spectrophotometer. Fluorescence lifetimes were measured by Fluorolog 3–11 (HORIBA Jobin Yvon). Absolute fluorescence quantum yield of the CDs was determined by a Fluoromax-4 measurement system (HORIBA, JobinYvon. Inc).

### Preparation of NIR-CDs

The CDs were synthesized via a one-step microwave-assisted carbonization manner [36, 59]. In brief, reduced glutathione (0.5 g) was dissolved in 20 mL formamide under ultrasonic treatment for 5 min. Subsequently, the mixture was transferred into a domestic microwave oven (700 W) for 3 min. After being cooled down to room temperature naturally, the obtained dark green solution was centrifuged at 10,000 rpm for 5 min to remove large-sized nanoparticles, and purified by a dialysis of 5 days (cut-off molecular weight, 3500). Then, the CDs solution was dried by a rotary evaporation to remove water. Finally, the product i.e., dark green NIR-CDs powder was harvested.

### Isolation of chloroplasts

Chloroplasts were extracted from fresh leaves of tobacco that planted in our lab. The procedures were performed according to the previous literature [38]. Briefly, 5 g fresh leaves were cleaned by water and cut into small pieces, and then transferred into sucrose buffer (0.4 M sucrose, 0.03 M Na_2_HPO_4_, 0.02 M KH_2_PO_4_ and 0.01 M KCl, pH 7.7). After a quick grind of 5 min, the filtrate was centrifuged at 1000 rpm for 3 min to remove large residues. And the supernatant was collected and centrifuged at 3000 rpm for 3 min. Finally, the precipitate was collected and re-dispersed in 5 mL sucrose buffer to obtain a chloroplast suspension with the concentration calculated to be ca. 2.0 mg/mL. All the operations were carried out in the dark at 0–4 °C.

### Fabrication of the CDs/chloroplast complex

To acquire the complex of CDs-chloroplast, a simple mix between NIR-CDs and chloroplast suspension was performed in the sucrose buffer for 0.5 h at 4 °C. The CDs would interact with chloroplasts, and were adsorbed onto the surface of chloroplasts and then form the CDs/chloroplast complexes.

### Plant cultivation and NIR-CDs treatment

*Nicotiana benthamiana* (*N. benthamiana*) seeds were germinated in dark and moist conditions for 3 days. The germinated seeds were transferred into planting cups and cultured in a greenhouse (25 ± 2 °C, 60% relative humidity, 400 μmol/m/s light intensity, 16 h light/8 h dark photoperiod). Then, seedlings in six-leaf stage showing consistent growth were selected and uniformly divided into five groups. NIR-CDs solutions at 0.01 mg/mL, 0.05 mg/mL, 0.1 mg/mL, 0.3 mg/mL were sprayed on seedlings with a dosage of 20 mL/pot on the second day after transplantation, and 20 ml of ultrapure water was sprayed as the control. Phenotypic changes were observed and recorded for the next 5 days. Each treatment was repeated three times with ten plants.

### Growth potential index determination

The root length, stem length, leaf area and single plant fresh weight (FW) of 10 N*. benthamiana* seedlings from each concentration of NIR-CDs treatment were measured after 5 days. Fresh weight was determined by gravimetric method. Growth rate of single plant fresh weight was expressed as percent (%) = (single plant fresh weight of treatment group sample − single plant fresh weight of control group sample)/single plant fresh weight of control group sample × 100%. The top two leaves of 10 seedlings from each treatment were collected for the measurement of leaf area.

The activity of superoxide dismutase (SOD) was determined according to our previous report [40] by following the photo-reduction of nitroblue tetrazolium (NBT) at 560 nm.

### Uptake and translocation of NIR-CDs visualized by laser confocal

Laser confocal images were obtained from *N. benthamiana* seedlings after 5 days of treatment to track the fluorescence of CDs. The complete part of the root tip, blade back of leaves without veins, and the transection of stem were selected for preparing seedling tissue sections by hand-slicing. Then the tissue sections were placed into clean glass slides, followed by covering with a cover slip for fixation. Subsequently, thin slices of different parts (root, stem and leaf) of the plants were observed by Leica laser scanning confocal microscopy (LEICA TCS SP8, GER) excited at 488–587 nm to identify the locations of NIR-CDs [50].

### Photosynthetic efficiency and pigment measurement

*N. benthamiana* seedlings treated with 0.05 mg/mL of NIR-CDs solution were sampled and determined in seven-leaf, ten-leaf, and thirteen-leaf stage, respectively. Net photosynthetic rate (Pn), transpiration rate (Tr), stomatal conductance (Cond), and mesophyll intercellular CO_2_ (Ci) were measured using LI-6400 portable photosynthesis meter (Li-COR company, USA) according to a reported method [40]. The total chlorophyll (Chl a + b) contents of *N. benthamiana* leaves were measured using SPAD (Special Products Analysis Division)-502^®^ plus chlorophyll meter (Minolta Camera Co., Osaka, Japan).

### Quantitative qPCR analysis of the genes involved in photosynthesis

Based on the above photosynthetic physiological indexes, NIR-CDs solutions at 0.05 mg/mL was the optimal treatment concentration. After a treatment of 5 days, the leaves of 0.05 mg/mL NIR-CDs treatment were frozen quickly with liquid nitrogen, and the seedlings sprayed with ultrapure water were used as control. The total RNA in these leaves was extracted and reverse-transcribed to cDNA using a Plant Total RNA Reagent (Invitrogen) and TransScript All-in-One First-Strand cDNA Synthesis SuperMix for qPCR (TransGen Biotech, Beijing, China) following the manufacturer’s protocol, respectively. The genes expression levels of *PSI-K* (AY899937.1) in photosystem I, *psbP* (KF460578.1), *psbS1* (EU645483.1), *psbY* (EH369921.1), *HCF136* (EH364276.1) in photosystem II, and *psbQ1* (JF897611.1), *psbQ2* (JF89761 2.1), *psbO4* (JF897606.1) for precursors of chloroplasts synthesis were measured by quantitative real-time (qRT)-PCR analysis using *GAPDH* as an internal reference. The genes sequences were retrieved from National Center for Biotechnology Information (https://www.ncbi.nlm.nih.gov/) to design primers (Table 1) using Primer Premier software, version 5. Each reaction contained 1 μL of cDNA, 5 μL of 2 × Power SYBR green PCR master mix (Applied Biosystems, Forster City, CA, USA), and 1 μL of forward and reverse primers in a final volume of 10 μL. Finally, the relative gene expression level was calculated using the 2^−ΔΔCT^ method. Dissociation-curve analysis was carried out to confirm the amplification specificity. Three technical replicates were performed for each sample.

### Statistical analysis

All experiments results were expressed as means ± standard deviation (SD). Statistical significance of all data was determined using a one-way analysis of variance (ANOVA) and the significance between treatments was assessed by LSD multiple comparison test at *P* < 0.05.

## Supplementary Information

**Additional file 1: Figure S1.** Raman spectrum of the NIR-CDs. **Figure S2.** High resolution XPS spectra of C 1 s (a), N 1 s (b), O 1 s (c) and S2p (d), respectively. **Figure S3.** Zeta potential measurement of the NIR-CDs. **Figure S4.** FL decay fitting curve of the NIR-CDs (10 µg/mL) in water (λ_ex_ = 420 nm, λ_em_ = 680 nm). **Figure S5.** Photostability measurement of the CDs (10 µg/mL, relative intensities recorded at 680 nm) under continuous UV-light irradiation. **Figure S6.** The storage stability assessment of the CDs (100 µg/mL) in aqueous solution. **Table S1.** A detailed comparison on optical properties, toxicity and mechanism between this work and previous reports.

## Data Availability

The genes sequences of *N. benthamiana* were retrieved from National Center for Biotechnology Information (https://www.ncbi.nlm.nih.gov/). Complementary data of CDs are supplied as Additional file 1.
