# Supplementary material for: Fluorescent carbon-dots enhance light harvesting and photosynthesis by overexpressing PsbP and PsiK genes
Source: J Nanobiotechnology. 2021 Aug 28;19:260. doi: 10.1186/s12951-021-01005-0 (PMC8403421; doi:10.1186/s12951-021-01005-0)
Supplement: Supplementary file 1 — Additional file 1: Figure S1. Raman spectrum of the NIR-CDs. Figure S2. High resolution XPS spectra of C 1 s (a), N 1 s (b), O 1 s (c) and S2p (d), respectively. Figure S3. Zeta potential measurement of the NIR-CDs. Figure S4. FL decay fitting curve of the NIR-CDs (10 µg/mL) in water (λex = 420 nm, λem = 680 nm). Figure S5. Photostability measurement of the CDs (10 µg/mL, relative intensities recorded at 680 nm) under continuous UV-light irradiation. Figure S6. The storage stability assessment of the CDs (100 µg/mL) in aqueous solution. Table S1. A detailed comparison on optical properties, toxicity and mechanism between this work and previous reports. [file 12951_2021_1005_MOESM1_ESM.docx]

Additional file 1

**Fluorescent carbon-dots for enhanced light harvesting and photosynthesis by overexpressing PsbP and PsiK genes**

Y Yuhui Wang^1†^, Zhuomi Xie^2,3†^, Xiuhua Wang^1^, Xin Peng^2*^, Jianping Zheng^1*^

1 Cixi Institute of Biomedical Engineering, Ningbo Institute of Materials Technology & Engineering, Chinese Academy of Sciences, Ningbo 315300, P. R. China.

2 Ningbo Research Institute of Zhejiang University, Ningbo, 315100, P. R. China.

3 Fujian Agriculture and Forestry University, Fuzhou, 350028, P. R. China.

**†**Yuhui Wang and Zhuomi Xie have contributed equally to this work

***Correspondence:** pengx@nit.zju.edu.cn; zhengjianping@nimte.ac.cn

**Figure S1**. Raman spectrum of the NIR-CDs.

**Figure S2**. High resolution XPS spectra of C 1s (a), N 1s (b), O 1s (c) and S2p (d), respectively.

**Figure S3**. Zeta potential measurement of the NIR-CDs.

**Figure S4**. FL decay fitting curve of the NIR-CDs (10 µg/mL) in water (λ_ex_ = 420 nm, λ_em_ = 680 nm).

**Figure S5**. Photostability measurement of the CDs (10 µg/mL, relative intensities recorded at 680 nm) under continuous UV-light irradiation.

**Figure S6**. The storage stability assessment of the CDs (100 µg/mL) in aqueous solution.

**Table S1**. A detailed comparison on optical properties, toxicity and mechanism between this work and previous reports.

| **Luminescent Materials** | **Abs (nm)** | **Emission (nm)** | **Fresh weight enhancement** | **Toxicity** | **Mechanism** | **Ref** |
| --- | --- | --- | --- | --- | --- | --- |
| CdTe/CdS/ZnS-QDs | 350-450 | 570-585 | Not mentioned | Y | Not mentioned | S1 |
| silicon QDs | 300-400 | 400-550 | 40.6% | N | Enzyme level | S2 |
| up-conversion phosphors | 980 | 450-480, 520-560, 650-675 | 57.3% | Y | Not mentioned | S3 |
| metal nanoclusters | 300-400 | 400-500 | 12.2% | Y | Gene and enzyme level | S4 |
| CDs | 300-450 | 400-600 | 34.9% | N | Enzyme level | 55 |
| PVA@CDs Nanocapsule | 300-400 | 400-600 | 26.45% | N | Not mentioned | S6 |
| CDs | 350-550 | 480-640  650-750 | Not mentioned | N | Enzyme level | S7 |
| Far-red CDs | 300-450 | 625-800 | 51.14% | N | Not mentioned | S8 |
| NIR-CDs | 300-450 | 625-710 | 247.03% | N | Gene and enzyme level | This work |

**Reference**

S1. S. Lin, P. Bhattacharya, N. C. Rajapakse, D. E. Brune, P. C. Ke. *J. Phys. Chem. C.*, 2009, 113, 10962–10966.

S2. Y. Li, W. Li, H. Zhang, Y. Liu, L. Ma, B. Lei. *Nanoscale*, 2020, 12, 155-166.

S3. J. Peng, Y. Sun, Q. Liu, Y. Yang, J. Zhou, W. Feng, X. Zhang, F. Li. *Nano. Res*., 2012, 5, 770–782.

S4. C. Wang, X. Liu, J. Li, L. Yue, H. Yang, H. Zou, Z. Wang, B. Xing. *Environ. Pollut*., 2021, 289, 117912.

S5. H. Wang, M. Zhang, Y. Song, H. Li, H. Huang, M. Shao, Y. Liu, Z. Kang. *Carbon*, 2018, 136, 94-102.

S6. X. Xu, X. Mao, J. Zhuang, B. Lei, Y. Li, W. Li, X. Zhang, C. Hu, Y. Fang, Y. Liu. *ACS Sustainable. Chem. Eng*., 2020, 8, 9, 3938–3949.

S7. W. Li, S. Wu, H. Zhang, X. Zhang, J. Zhuang, C. Hu, Y. Liu, B. Lei, L. Ma, X. Wang. *Adv. Funct. Mater*., 2018, 1804004.

S8. D. Li, W. Li, H. Zhang, X. Zhang, J. Zhuang, Y. Liu, C. Hu, B. Lei. *ACS Appl. Mater. Interfaces*., 2020, 12, 21009−21019.
